# A Randomized Trial of the Effects of Dietary n3-PUFAs on Skeletal Muscle Function and Acute Exercise Response in Healthy Older Adults

**DOI:** 10.3390/nu14173537

**Published:** 2022-08-27

**Authors:** Hawley E. Kunz, Kelly L. Michie, Kevin J. Gries, Xiaoyan Zhang, Zachary C. Ryan, Ian R. Lanza

**Affiliations:** 1 Endocrine Research Unit, Division of Endocrinology, Department of Internal Medicine, Mayo Clinic, Rochester, MN 55905, USA; 2 Department of Physical Therapy, School of Health Professions, Concordia University of Wisconsin, Mequon, WI 53097, USA; 3 Department of Geriatrics, Shanghai Jiaotong University Affiliated Sixth People’s Hospital, Shanghai 200233, China

**Keywords:** n3-PUFA, aging, exercise, anabolic resistance

## Abstract

Skeletal muscle is critical for maintaining mobility, independence, and metabolic health in older adults. However, a common feature of aging is the progressive loss of skeletal muscle mass and function, which is often accompanied by mitochondrial impairments, oxidative stress, and insulin resistance. Exercise improves muscle strength, mitochondrial health, and cardiorespiratory fitness, but older adults often exhibit attenuated anabolic responses to acute exercise. Chronic inflammation associated with aging may contribute to this “anabolic resistance” and therapeutic interventions that target inflammation may improve exercise responsiveness. To this end, we conducted a randomized controlled trial to determine the effect of 6 months of dietary omega-3 polyunsaturated fatty acids (n3-PUFA) supplementation on skeletal muscle function (mass, strength), mitochondrial physiology (respiration, ATP production, ROS generation), and acute exercise responsiveness at the level of the muscle (fractional synthesis rate) and the whole-body (amino acid kinetics) in healthy older adults. When compared with a corn oil placebo (*n* = 33; 71.5 ± 4.8 years), older adults treated with 4 g/day n3-PUFA (*n* = 30; 71.4 ± 4.5 years) exhibited modest but significant increases in muscle strength (3.1 ± 14.7% increase in placebo vs. 7.5 ± 14.1% increase in n3-PUFA; *p* = 0.039). These improvements in muscle strength with n3-PUFA supplementation occurred in the absence of any effects on mitochondrial function and a minor attenuation of the acute response to exercise compared to placebo. Together, these data suggest modest benefits of dietary n3-PUFAs to muscle function in healthy older adults. Future studies may elucidate whether n3-PUFA supplementation improves the exercise response in elderly individuals with co-morbidities, such as chronic inflammatory disease or sarcopenia.

## 1. Introduction

The rapid growth of the aging population is motivating the scientific and healthcare communities to identify new ways to help prolong human healthspan, or the length of time a person remains healthy and free from debilitating disease. Toward this goal, tremendous attention has been centered on skeletal muscle because of its critical role in mobility, independence, metabolic health, and overall well-being. A common feature of aging is a progressive loss of skeletal muscle mass and function (i.e., sarcopenia) [1,2], which is associated with physical disability, loss of independence, poor quality of life, increased risk of falls, and overall morbidity and mortality [3,4]. Sarcopenia is often accompanied by mitochondrial impairments [5], oxidative stress [6], and insulin resistance [7], which can lead to further deterioration of muscle health and metabolic disease. Exercise improves muscle strength [8], mitochondrial health [9], cardiorespiratory fitness [10,11], and longevity [12,13] and remains a frontline strategy to maintain skeletal muscle health in older adults. Although exercise promotes good health regardless of age, older adults often exhibit attenuated anabolic responses to acute exercise or nutritional stimuli [5,14,15]. Ergogenic aids may have some benefit to older adults in helping them overcome this so-called “anabolic resistance” and maximize the therapeutic benefit of exercise prescriptions.

Several targetable pathways have emerged in the quest to understand the mechanisms of age-related anabolic resistance. Underlying mechanisms include altered anabolic signaling pathways [16], mitochondrial dysfunction [17], and accumulation of senescent cells during critical periods of muscle growth and repair [18,19]. Indeed, the accumulation of senescent cells and pro-inflammatory immune cells are believed to contribute to the state of chronic, low-grade inflammation that is a hallmark of aging [20,21,22]. Further evidence that chronic inflammation may play an important role in age-related anabolic resistance comes from earlier observations that elevated levels of tumor necrosis factor α (TNFα) are associated with reduced muscle protein synthesis [23,24], and older individuals with increased levels of *C*-reactive protein (CRP) exhibit heightened proteasomal activity [20]. This evidence base fueled several initiatives to evaluate how anabolic response to exercise could be enhanced by targeting age-related inflammation through various approaches including senolytic agents [18,19], non-steroidal anti-inflammatory drugs [25], and dietary omega-3 fatty acids [5,26,27].

Dietary long-chain omega-3 polyunsaturated fatty acids (n3-PUFA) are widely recognized for their triglyceride-lowering effects [28], notable anti-inflammatory properties [29,30,31,32], and minimal side-effects and health risks. These features make n3-PUFAs enticing agents to potentially enhance anabolic responses to exercise in older adults by reducing inflammatory signals that may attenuate appropriate anabolic signaling in skeletal muscle with exercise. One study found that 8 weeks of dietary supplementation with 1.86 g/day eicosapentaenoic acid (EPA) and 1.5 g/day docosahexaenoic acid (DHA), two abundant n3-PUFAs in marine oils, enhanced the stimulatory effect of insulin and amino acids on muscle protein synthesis [26]. Another study found that n3-PUFAs enhanced the strength gains with exercise in older women [33]. Building upon these studies, we recently conducted an open-label, single-arm study in a small cohort of older adults where we found that 3.9 g/day of dietary n3-PUFA for 16 weeks enhanced the effects of acute resistance exercise in muscle protein synthesis [5]. In this cohort, we also observed a reduction in mitochondrial reactive oxygen species (ROS) production following n3-PUFA supplementation [5], suggesting that the beneficial effects of n3-PUFAs on skeletal muscle may be partially mediated through reduced oxidative stress and improved mitochondrial function. Preclinical studies in aged mice showed EPA supplementation enhanced mitochondrial oxidative capacity in skeletal muscle [34].

Identifying therapeutic strategies for improving muscle mass, function, and anabolic responses to exercise in the elderly is a high priority in the quest to maintain physical function and prolong healthspan in older adults. While early evidence suggests that n3-PUFA supplementation may be an effective, easy-to-implement strategy for improving muscle health and exercise responsiveness, concrete recommendations for older adults await rigorous human trials to evaluate the effects of n3-PUFAs on anabolic responses to acute exercise and mitochondrial function. We conducted a randomized controlled trial to determine the effect of 6 months of EPA + DHA (~4 g/day) supplementation on skeletal muscle function, mitochondrial physiology, and acute exercise responsiveness in older adults. When compared with placebo, older adults treated with EPA + DHA exhibited modest but significant increases in muscle strength. The improvements in muscle strength occurred in the absence of any clear effects on whole-body or muscle-specific protein metabolism or mitochondrial function. Together, these data suggest modest benefits of dietary n3-PUFAs to muscle function in healthy older adults.

## 2. Materials and Methods

### 2.1. Participants and Study Design

**Figure 1:**
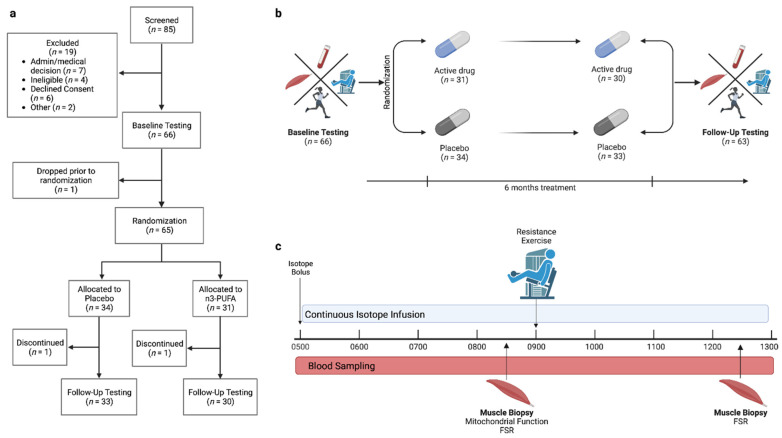
Participant enrollment and study design. (**a**) Enrollment numbers and flow chart for the study. (**b**) Overall study design of the intervention, which included baseline and follow-up assessments of physical activity, cardiorespiratory fitness, muscle strength and function, mitochondrial physiology, body composition, and whole-body and mixed muscle protein synthesis. All assessments were completed before and after 6 months of placebo or n3-PUFA supplementation, administered in a randomized, double-blind design. (**c**) Study design for the inpatient study day. Figure created using BioRender.com.

Sixty-three older adults (65–85 years, 71.4 ± 4.6 years; 29 male/34 female) from Rochester, Minnesota and the surrounding areas completed the study (Figure 1a) from January 2017 to December 2021. Participants were independently living adults without a known chronic debilitating disease. Participants were excluded if they participated in more than 30 min of structured physical activity or training 3 times per week or more. Exclusion criteria included regular n3-PUFA supplementation, anemia (hemoglobin < 11 g/dL for females and <12 g/dL for males), diabetes or fasting plasma glucose > 126 mg/dL, active coronary artery disease, renal insufficiency (serum creatinine > 1.5 mg/dL), liver disease (aspartate aminotransferase [AST] > 144 IU/L or alanine aminotransferase [ALT] > 165 IU/L), blood clotting disorders, impaired coagulation (international normalized ratio [INR] > 2.0), cigarette or tobacco use, alcohol or other substance abuse, untreated or uncontrolled thyroid disease, or fish or shellfish allergy. Participants taking any medication that may influence the outcomes or increase the risks of the study (e.g., metformin, insulin, tricyclic antidepressants, benzodiazepines, opiates, barbiturates, anticoagulants) were also excluded. Study participation was comprised of a screening visit, an outpatient testing visit and an overnight inpatient visit, detailed below. Following the overnight inpatient visit, participants were randomly assigned to receive either placebo or n3-PUFA. After 6 months on the assigned intervention, all outpatient and inpatient testing was repeated (Figure 1b). The study was registered under Clinical Trial Number NCT03350906, and study procedures were approved by the Mayo Foundation Institutional Review Board (IRB 17-004403) and conformed to the principles of the Declaration of Helsinki.

### 2.2. Screening Visit

Interested participants completed an initial screening questionnaire by phone or email. After initial screening, interested and eligible participants reported to the Mayo Clinic Clinical Research and Trials Unit (CRTU) for consent and additional screening. The procedures and risks associated with the study were discussed with the participants. All enrolled participants provided written informed consent. Participant weight, height, pulse, and blood pressure were measured and recorded by a registered nurse. A fasting, antecubital blood sample was collected to evaluate participant eligibility and to assess circulating markers of inflammation. From the blood samples, the Mayo Clinic Laboratories performed a complete blood count (CBC) with differential and biochemical tests of glucose, insulin, ALT, AST, prothrombin time, INR, creatinine, thyroid-stimulating hormone, erythrocyte sedimentation rate (ESR), and high-sensitivity CRP. To assess free-living physical activity, participants were given a 3-axis, hip-worn accelerometer (wGT3X-BT, Actigraph, Pensacola, FL, USA) and were asked to wear the monitor during all waking hours for a period of 2 weeks. A sampling frequency of 30-Hz was used, and data were processed and analyzed in 60-s epochs using ActiLife software (v6.9.5, Actigraph, Pensacola, FL, USA). Days of valid activity monitoring comprised a minimum of 10 h of wear-time, assessed using the Choi et al. [35] method. Average step counts per day and the average daily number of minutes spent in moderate to vigorous physical activity (MVPA), as defined by Freedson et al. [36], was determined for 7 valid days, including 2 weekend days.

### 2.3. Outpatient Testing Visit

Approximately 4 weeks after the screening visit, participants returned to the CRTU for additional testing. Body composition was determined using dual-energy X-ray absorptiometry (DEXA; GE Lunar iDXA, GE Healthcare, Chicago, IL, USA). Peak whole-body oxygen consumption (VO_2_peak) was assessed by indirect calorimetry during a graded treadmill test using either the Bruce maximal protocol [37] or the Modified Bruce maximal protocol [38], as described previously [39]. Single-leg knee extensor strength was measured on a pneumatic resistance leg extension machine (Keiser Air300, Keiser Corporation, Fresno, CA, USA) and defined as the unilateral 1-repetition maximum (1-RM). Participants were habituated to the unilateral knee extension exercise and performed a warm-up consisting of 10 repetitions at minimal resistance and 3 sets of 5–10 repetitions at increasing workloads as determined by the investigator. Participants then performed a series of single-repetition attempts at increasing workloads, each separated by 3 min of rest, until the maximum load that could be moved through the entire range of motion was reached. This maximum workload was defined as the individual 1-RM [39,40,41]. After determining 1-RM, peak single-leg knee extensor power was evaluated at 70% of the individual 1-RM, as previously described [40,41]. Briefly, after 5 min of rest, participants performed the unilateral knee extension as quickly as possible through the full range of motion. Participants performed 3–5 single repetitions, with each individual repetition separated by 30 s. The highest measured power was recorded as the peak power. After 5 min of rest, local muscle endurance was determined by the number of consecutive repetitions through the full range of motion that each participant could perform at 70% of the individual 1-RM [42].

### 2.4. Inpatient Study Day

The inpatient study day (Figure 1c) was scheduled between 1 week and 3 months following the outpatient testing visit. For the 3 days preceding the inpatient study day, weight-maintaining meals were provided to study participants by the Mayo Clinic Metabolic Research Kitchen (food preferences and dietary restrictions were discussed with a dietitian from the Research Kitchen during the outpatient study visit). The diet comprised ~20% protein, ~50% carbohydrate, and ~30% fat, with caloric needs to maintain weight determined using the Harris Benedict Equation [43]. On the evening before the inpatient study day, participants reported to the CRTU at approximately 1730 h. Following an evening meal at 1800 h, participants remained fasted but were given water ad libitum. At 400 h the following morning, a peripheral intravenous catheter was placed for infusion of stable isotope tracers. The opposite hand rested in a plexiglass box maintained at 55 °C for the collection of arterialized venous blood samples from a heated retrograde intravenous catheter. Blood sampling and isotope infusion began at 500 h. At 830 h, a percutaneous muscle biopsy was performed on the vastus lateralis muscle using a modified Bergstrom needle and local anesthetic (2% lidocaine) [44]. Fresh muscle tissue was rapidly prepared bedside for muscle fiber permeabilization and subsequent mitochondrial functional assessments. The remaining biopsy tissue was snap-frozen in liquid nitrogen and maintained in storage at −80 °C for subsequent measurements of muscle protein fractional synthesis rates (FSR). After the first muscle biopsy (~900 h), participants completed 8 sets of 10 single leg extensions on the contralateral leg at the resistance corresponding to 70% of the individual’s 1-RM, measured on the same leg during the outpatient study visit. A muscle biopsy was performed on the exercised vastus lateralis muscle 3 h after the cessation of exercise (~1230 h) and frozen in liquid nitrogen for FSR measurements.

### 2.5. Intervention

Upon completion of the inpatient study day, participants were randomly assigned to receive either placebo or n3-PUFA based on a statistician-prepared randomization table. Investigators and participants were masked in the group assignments, and the Mayo Clinic Research Pharmacy oversaw medication procurement, storage, and dispensation in accordance with the randomization table. Upon study completion, established unblinding protocols were conducted by the Mayo Clinic IRB and the research pharmacy staff. Thirty-one adults were randomly assigned to the n3-PUFA group and instructed to swallow 2 softgels containing omega-3 fatty acid ethyl esters with morning meals and 2 softgels with evening meals for a period of 6 months. Each softgel (sold commercially as Ocean Blue^®^ Professional Omega-3 2100™) contained an orange flavoring and comprised a 1000-mg capsule with ~675 mg EPA (20:5*n*–3) and ~300 mg DHA (22:6*n*–3), providing a total dosage of 3.9 g n3-PUFA/d. Thirty-four older adults were randomly assigned to the placebo group and instructed to swallow two 1000-mg corn oil softgels, identical in appearance to the n3-PUFA softgels and containing the same orange flavoring, with morning and evening meals for 6 months. A 30-day supply of softgels was dispensed by the research pharmacy upon completion of the inpatient study day. Participants subsequently returned to the CRTU every 4 weeks for the next 6 months to obtain a refill of the study medication and for measurement of blood coagulation and liver function. Participants were instructed to return any unused medication to the research pharmacy, and the remaining softgels were counted to assess compliance. Compliance was also assessed by measuring EPA and DHA concentrations in red blood cells before and after the intervention, as previously described [28]. Briefly, blood collected in 4 mL EDTA vacutainers was centrifuged at 650× *g* and 4 °C for 10 min. The plasma and buffy coat were removed, and the packed erythrocytes were washed twice with cold 0.9% NaCl and frozen at −80 °C. The concentrations of EPA and DHA in the red blood cells were measured on a triple quadrupole mass spectrometer coupled with an ultra-pressure liquid chromatography system (LC/MS) and calculated against a standard curve, as previously described [28].

### 2.6. Mitochondrial Measurements in Permeabilized Muscle Fibers

Approximately 30 mg of muscle tissue was used for mitochondrial measurements. Fresh tissue was dissected into two 8–12 mg sections for measurement of mitochondrial respiration and reactive oxygen species (ROS) production and two 4.5–8 mg sections for measurement of ATP production. Muscle sections were dissected and weighed before being placed in cold relaxing and preservation BIOPS buffer (10 mM Ca-EGTA buffer, 0.1 μM free calcium, 20 mM imidazole, 20 mM taurine, 50 mM K-MES, 0.5 mM DTT, 6.56 mM MgCl_2_, 5.77 mM ATP, 15 mM phosphocreatine, pH 7.1) [45]. Fibers were mechanically separated longitudinally and then chemically permeabilized with saponin (50 μg/mL) as previously described [46,47,48]. Duplicate sets of fibers were prepared and added to the dual chambers of an Oxygraph high resolution respirometer (Oroboros Instruments, Innsbruck, Austria) containing, MiR05 respiration buffer (0.5 mM EGTA, 3 mM MgCl_2_·6H_2_O, 60 mM potassium lactobionate, 20 mM taurine, 10 mM KH_2_PO_4_, 20 mM HEPES, 110 mM Sucrose, and 1 g/L fatty acid free BSA) [49]. The oxygen concentration within the chambers was maintained between 200 and 400 μM throughout respiration measurements, and chambers were supplemented with blebbistatin (25 μM) to inhibit fiber contraction [50]. Oxygen consumption (*J*O2) was measured continuously using a stepwise protocol that involved the sequential addition of substrates and inhibitors to assess State 1 respiration, State 2 respiration (10 mM glutamate, 2 mM malate, 10 mM succinate) and State 3 respiration through complex I and II (5 mM ADP) [49]. The acceptor control ratio (ACR: State 3/State 2) was calculated for each sample. Mitochondrial ROS production (H_2_O_2_ flux) rates were multiplexed with measurements of oxygen consumption using the Oxygraph O2K-Fluorescence LED2-Module to assess the oxidation of Amplex Red, as previously described [51].

ATP production (*J*ATP) was assessed in duplicate using a spectrofluorometer (Fluorolog 3, Horiba) in an additional set of permeabilized muscle fibers, as described previously [52,53,54]. Briefly, buffer Z (110 mM K-MES, 35 mM KCl, 1 mM EGTA, 5 mM K_2_HPO_4_, 3 mM MgCl_2_·6H2O, 5 mg/mL BSA; pH 7.4, 295 mOsm) was supplemented with D-glucose (2.5 mM), hexokinase (2.5 U/mL), glucose-6-phosphate dehydrogenase (2.5 U/mL), and NADP+ (2 mM) to catalyze the conversion of ATP to NADPH. P1,P5-Di(adenosine-5)pentaphosphate (100 µM) was added to eliminate interference by residual adenylate kinase activity. The NADPH autofluorescence was continuously monitored as a surrogate indicator of ATP production using the same stepwise protocol described for the measurement of *J*O_2_. MATLAB (MathWorks) was used to calculate *J*ATP from the fluorescence signal.

*J*O_2_ and *J*ATP were normalized to wet tissue weight. H_2_O_2_ flux rates were normalized to wet tissue weight and, to evaluate ROS production beyond that produced during normal respiration, to oxygen consumption at the corresponding respiratory state.

### 2.7. Stable Isotope Tracer Administration

Whole-body protein turnover and mixed muscle protein fractional synthesis rates (FSR) were assessed with the use of stable isotope tracers (Cambridge Isotopes Inc., Cambridge, MA, USA). At 0500 h on the inpatient study day, 1 mg/kg fat free mass (FFM) [^13^C_6_]phenylalanine, 0.5 mg/kg FFM [^13^C_6_]tyrosine, and 0.5 mg/kg FFM [^15^N]tyrosine were administered as priming boluses through the peripheral intravenous catheter. A continuous infusion of [^13^C_6_]phenylalanine (1 mg/kg FFM/h) and [^15^N]tyrosine (0.5 mg/kg FFM/h) was immediately initiated and maintained for 8 h.

### 2.8. Whole-Body Protein Turnover

Blood samples were obtained before the isotope bolus injections and at 14 time points during the continuous infusion (6 pre-exercise; 8 post-exercise). Isotopic enrichments (IE) of [^13^C_6_]phenylalanine, [^13^C_6_]tyrosine, and [^15^N]tyrosine were measured in plasma by gas chromatography/mass spectrometry (GCMS), as previously described [55].

After achieving isotopic steady-state, the average plasma IEs of [^13^C_6_]phenylalanine, [^13^C_6_]tyrosine, and [^15^N]tyrosine were used to calculate whole-body amino acid kinetics using the single-pool steady-state model [56,57]. Amino acid kinetics were calculated for the steady-state achieved in the 6 blood draws pre-exercise and for the steady-state achieved in the 8 blood draws post-exercise. Whole-body [^13^C_6_]phenylalanine and [^15^N]tyrosine flux (*Q_flux_*), were calculated from the equation:

$$Q_{flux}=i*\;\left(\frac{E_{i}}{E_{p}}-1\right)$$

where *i* is the infusion rate, *E_i_* is the moles percent excess (MPE) of the isotope in the infusate, and *E_p_* is the MPE in the plasma. Because all measurements were performed during isotopic steady state, *Q_flux_* is assumed to represent both the rate of isotope appearance and disappearance.

The oxidation rate of phenylalanine (*I_PT_*) was estimated by the conversion rate of phenylalanine to tyrosine using the equation:

$$I_{PT}=Q_{T}*\;\left(\frac{13C_{6}Tyr_{Ei}}{13C_{6}Phe_{Ei}}\right)*\left(\frac{Q_{P}}{I_{P}+Q_{P}}\right)\;$$

where *Q_T_* and *Q_P_* are the [^15^N]tyrosine and [^13^C_6_]phenylalanine fluxes, respectively; [^13^*C*_6_]*Tyr_Ei_* and [^13^*C*_6_]*Phe_Ei_* are the plasma enrichments of [^13^C_6_]tyrosine and [^13^C_6_]phenylalanine, respectively; and *I_P_* is the infusion rate of [^13^C_6_]phenylalanine.

The conversion of phenylalanine to protein (*Q_PS_*), which represents whole-body protein synthesis, was estimated by subtracting the oxidation rate of phenylalanine from the phenylalanine flux:

$$Q_{PS}=Q_{P}-I_{PT}$$

Whole body protein balance (*PB*) was estimated by subtracting phenylalanine flux (a measure of protein breakdown) from the estimate of protein synthesis.

$$PB=Q_{PS}-Q_{P}$$

For all measurements of amino acid kinetics, delta values were calculated as the difference between pre- and post-exercise (Post-Exercise—Pre-Exercise).

### 2.9. Skeletal Muscle Fractional Synthesis Rates

The IE of [^13^C_6_]phenylalanine in mixed muscle protein (MMP) pools and in protein-free muscle tissue fluid (TF), measured by HPLC and tandem mass spectrometry, was used to calculate muscle fractional synthesis rates (FSR), as previously described [58,59,60]. Briefly, snap-frozen muscle biopsies were pulverized into a fine powder with a stainless-steel mortar and pestle maintained below −20 °C. Using 5% sulfosalicylic acid, TF free amino acids were extracted from ~30 mg of MMP powder. The remaining mixed muscle tissue was hydrolyzed in 6N HCL at 110 °C overnight. MMP and TF samples were then purified using cation exchange columns (AG 50W-X8 resin; Bio-Rad, Hercules, CA, USA), dried, and derivatized to isobutyl esters [5]. HPLC and tandem mass spectrometry data acquisition was performed in positive electrospray ionization mode and ion monitoring was selected at 222.4 > 121.6 and 226.4 > 125.6 for the m + 2 and m + 6 fragments of phenylalanine and [^13^C_6_]phenylalanine, respectively [5]. The MPE for MMP and TF was calculated against a 6-point enrichment standard curve. FSR was calculated with the equation:

$$FSR=\left(\frac{E_{p2}-E_{p1}}{E_{precursor}*t}\right)*100$$

where *E_p2_* and *E_p1_* are the MMP-bound enrichments of [^13^C_6_]phenylalanine in muscle biopsies collected at ~1230 h and ~830 h, respectively (Figure 1c). *E_precursor_* is the enrichment of [^13^C_6_]phenylalanine in TF or in the plasma (average enrichment after achieving steady state) to estimate the upper and lower limits, respectively, of the true FSR [60]. Tracer incorporation time (*t*) was the time in hours between the two biopsies.

### 2.10. Statistical Analysis

Data were assessed for normality of distribution and non-normal data was log-transformed. To assess baseline differences between groups (age, height), unpaired *t*-tests were performed. The effects of n3-PUFA were assessed using mixed repeated measures ANOVAs examining the within-subjects changes in time (baseline and follow-up), the between-subjects effects of the intervention (n3-PUFA or placebo), and the interaction between time and intervention. The effects of n3-PUFA supplementation on the whole-body amino acid response to exercise was evaluated using mixed repeated measured ANOVAs examining the within-subjects effects of exercise (pre- and post-exercise), the within-subjects effects of time (baseline and follow-up), the between-subjects effects of intervention (n3-PUFA or placebo) and the 2- and 3-way interactions between these variables. Pre-determined pairwise post hoc comparisons between pre- and post-intervention in each group were performed using the Sidak correction for multiple comparisons when significant main effects or interaction effects were observed. Significance was set a priori at *p* < 0.05. Statistical analyses was performed in Statistical Package for the Social Sciences (SPSS) v28 (IBM, Armonk, NY, USA). Power calculations were performed using the FSR data from our pilot cohort in which protein synthesis was measured using the same exercise protocol [5]. Using a linear random intercept sample size model with a baseline and a post intervention time point, with σ = 0.025%/h, a sample size of 30 participants per group yielded 80% power (α = 0.05) to detect a 0.028%/h slope change between groups in muscle protein synthesis in response to exercise.

## 3. Results

### 3.1. Participants and Intervention

Of the 65 participants randomly allocated to either placebo or n3-PUFA, a total of 2 participants (1 placebo, 1 n3-PUFA) were lost to follow up, leaving *n* = 33 in the placebo group and *n* = 30 in the n3-PUFA group (Figure 1a) for final analysis. Where data sets were incomplete or a loss of data occurred, the reduced *n*s are noted within the associated tables and figure legends. No supplement-related adverse events were reported, and liver enzymes (AST, ALT) and coagulation rates (prothrombin time and INR) remained within normal ranges for all participants throughout the 6-month intervention period. Unused pill counts indicated good participant compliance, and within the n3-PUFA group, the concentrations of DHA and/or EPA in RBCs increased in all participants in the active intervention group (Table 1). At baseline, the two groups were similar in age and body composition, and body composition did not significantly change in either group over the course of the intervention (Table 1). ESR and circulating CRP concentrations remained unchanged after the intervention in both groups.

### 3.2. Physical Activity and Function

**Figure 2:**
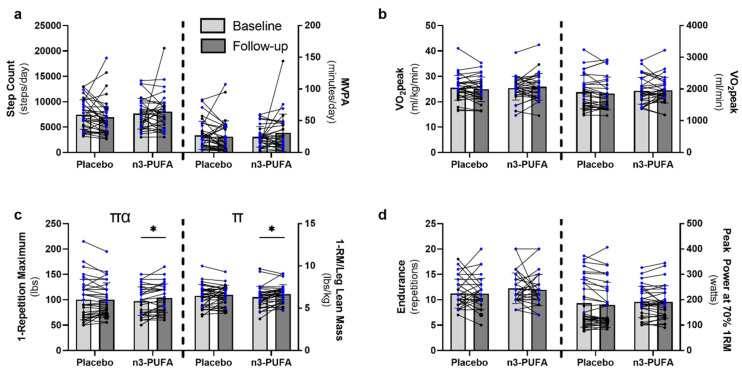
The effects of 6-months placebo or n3-PUFA on physical activity, cardiorespiratory fitness and skeletal muscle function. Free-living physical activity (**a**), cardiorespiratory fitness (**b**), and skeletal muscle strength (**c**), endurance, and power (**d**) were assessed in older adults (65–85 years) before and after 6 months of supplementation with either placebo or n3-PUFA. (**a**) Free-living physical activity was assessed by waist-worn accelerometers and defined as the number of steps per day or the number of minutes of moderate to vigorous physical activity (MVPA) per day (placebo: *n* = 33; n3-PUFA: *n* = 29). (**b**) Cardiorespiratory fitness (VO_2_peak) was determined with indirect calorimetry during a maximal graded treadmill test and expressed per kg body weight or as absolute oxygen consumption (placebo: *n* = 30; n3-PUFA: *n* = 30). (**c**) Single leg extension 1 repetition maximum (1-RM) was determined to assess muscle strength and is presented as absolute strength and as strength normalized to leg lean mass (placebo: *n* = 33; n3-PUFA: *n* = 30). (**d**) Single leg muscle endurance was defined as the maximum number of full repetitions that could be performed at 70% of the individual 1-RM, and single leg peak power was assessed at 70% of the individual 1-RM (placebo: *n* = 33; n3-PUFA: *n* = 30). Mixed repeated measures ANOVAs were performed to determine the within-subjects effects of time (baseline, follow-up), the between-subjects effects of the intervention (n3-PUFA, placebo) and the interaction between time and intervention. Post hoc pairwise comparisons were performed when significant main or interaction effects were identified. Significance was set a priori at *p* < 0.05. Individual data points represent individual participants, with blue data points representing males and black data points representing females. Bars indicate mean and standard deviation. π represents a significant main effect of time; α represents a significant time-by-intervention interaction; * represents a significant within-group difference between baseline and follow-up.

Free-living physical activity, as determined by number of steps and minutes of moderate to vigorous physical activity per day, was not different between the groups at baseline and did not change over the course of the intervention in either group (Figure 2a). Similarly, whole-body cardiorespiratory fitness did not differ between groups at baseline or post-intervention (Figure 2b). n3-PUFA supplementation better preserved and improved muscle strength over the course of the intervention (time-by-intervention interaction F = 4.457, *p* = 0.039) compared to placebo (Figure 2c). Within the n3-PUFA group, 1-RM strength improved significantly by an average of 7.5 ± 14.1%, ranging from a 5-lbs loss in strength to a 30-lbs increase. In contrast, the placebo group did not demonstrate significant improvements in 1-RM (3.1 ± 14.7%), with changes ranging from a 25-lbs loss to a 25-lbs gain in strength. A main effect of time (*p* = 0.019) was also observed when 1-RM was normalized to leg lean mass (Figure 2c). While the interaction between time and intervention was not significant (*p* = 0.182), post hoc pairwise comparisons demonstrated significant improvements only in the n3-PUFA group (*p* = 0.012), and not the placebo group (*p* = 0.443). n3-PUFA supplementation did not influence muscle endurance or power (Figure 2d). Taken together, these results show that n3-PUFA supplementation improved muscle strength in elderly individuals even without changes to daily physical activity.

### 3.3. Mitochondrial Function

**Figure 3:**
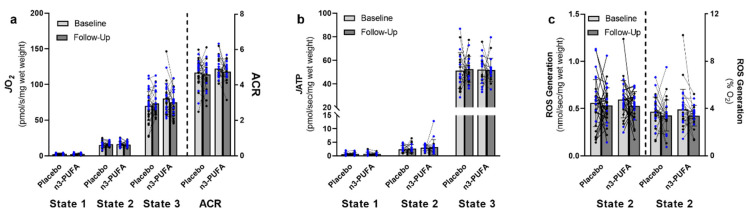
The effects of 6-months placebo or n3-PUFA on skeletal muscle mitochondrial function. Skeletal muscle mitochondrial function was evaluated by measuring (**a**) mitochondrial respiration (placebo: *n* = 30; n3-PUFA: *n* = 27), (**b**) ATP production (placebo: *n* = 20; n3-PUFA: *n* = 17), and (**c**) reactive oxygen species (ROS) generation (placebo: *n* = 30; n3-PUFA: *n* = 27) in permeabilized muscle fibers isolated from muscle biopsies of the vastus lateralis of older (65–85 years) adults before and after 6 months of placebo or n3-PUFA supplementation. (**a**,**b**) Respiration and ATP production were assessed at respiratory states 1 (baseline respiration), 2 (respiration in the presence of glutamate, malate, and succinate) and 3 (ADP-stimulated respiration through complex I + II) using an incremental, stepwise protocol. The acceptor control ratio (ACR) was calculated as the ratio of State 3:State 2 respiration. (**c**) ROS production was assessed during state 2 respiration. Oxygen (*J*O_2_) and ATP (*J*ATP) flux values were normalized to wet tissue weight and ROS generation was normalized to both wet tissue weight and to oxygen consumption at the corresponding respiratory state. Mixed repeated measures ANOVAs were performed to determine the within-subjects effects of time (baseline, follow-up), the between-subjects effects of the intervention (n3-PUFA, placebo) and the interaction between time and intervention. Significance was set a priori at *p* < 0.05. Individual data points represent individual participants, with blue data points representing males and black data points representing females. Bars indicate mean and standard deviation. *J*O_2_: oxygen flux; ACR: acceptor control ratio; *J*ATP: ATP flux; ROS: Reactive Oxygen Species.

Supplementation with n3-PUFA did not influence skeletal muscle mitochondrial function (Figure 3). Mitochondrial respiration (Figure 3a), ATP production (Figure 3b), and ROS generation (Figure 3c) were similar between the n3-PUFA and the placebo group at baseline, and these measurements did not significantly change over the course of the intervention in either group. While there was a modest trend for an overall reduction in ROS generation normalized to oxygen consumption over time (time effect F = 2.884, *p* = 0.095), pairwise post hoc comparisons did not find significant reductions in ROS generation in either the placebo (*p* = 0.376) or n3-PUFA group (*p* = 0.141).

### 3.4. Whole-Body Amino Acid Kinetics and Muscle Fractional Synthesis Rates

**Figure 4:**
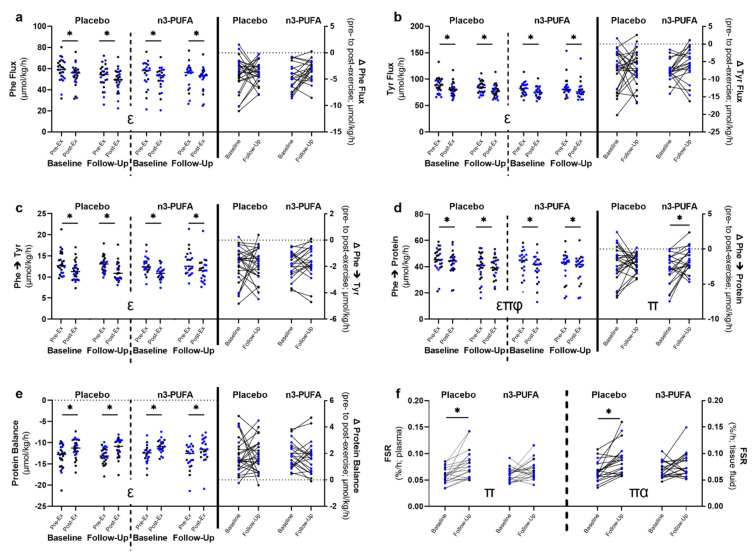
The effects of 6-months of placebo or n3-PUFA on whole-body amino acid kinetic and skeletal muscle fractional synthesis rate responses to acute exercise. (**a**–**e**) Whole-body amino acid kinetics were measured pre- and post-acute exercise (pre-ex; post-ex, respectively) in older (65–85 years) adults before (baseline) and after (follow-up) 6-months of placebo or n3-PUFA supplementation administered in randomized, double-blind fashion. Kinetics were determined using the triple tracer approach and assessed (**a**) phenylalanine (Phe) flux; (**b**) tyrosine (Tyr) flux; (**c**) the conversion of phenylalanine to tyrosine (Phe→Tyr); (**d**) the conversion of phenylalanine to protein (Phe→protein); and (**e**) the balance between protein synthesis and protein breakdown (placebo: *n* = 26; n3-PUFA: *n* = 21). Steady state was achieved before and after an acute bout of resistance exercise, and the absolute change in kinetics from pre- to post-exercise (Δ) was calculated (**a**-**e**). (**f**) Mixed skeletal muscle protein fractional synthesis rates associated with acute exercise were measured before and after 6 months of placebo or n3-PUFA supplementation. Acute exercise-associated mixed muscle protein fractional synthesis rates were calculated from the isotopic enrichment of labeled amino acid tracers measured in serial muscle biopsies collected before and after acute exercise (placebo: *n* = 20; n3-PUFA: *n* = 20). Plasma and tissue fluid isotopic enrichments were used as precursor pools to determine the low and high limits of the true FSR. For the whole-body amino acid kinetics, mixed repeated measures ANOVAs were performed to determine the within-subjects effects of time (baseline, follow-up) and exercise (pre-ex, post-ex), the between-subjects effects of the intervention (n3-PUFA, placebo) and the interactions between time, exercise, and intervention. For the Δ and FSR values, mixed repeated measures ANOVAs were performed to determine the within-subjects effects of time (baseline, follow-up), the between-subjects effects of the intervention (n3-PUFA, placebo) and the interaction between time and intervention. Significance was set a priori at *p* < 0.05. Post hoc pairwise comparisons were performed when significant main or interaction effects were identified. Individual data points represent individual participants, with blue data points representing males and black data points representing females. Bars indicate mean and standard deviation. ε represents a significant main effect of exercise; π represents a significant main effect of time; φ represents a significant exercise-by-time interaction; α represents a significant time-by-intervention interaction; * represents a significant within-group difference based on post hoc pairwise comparisons. Phe: phenylalanine; Tyr: tyrosine; FSR: fractional synthesis rate.

Whole-body protein turnover was assessed pre- and post-exercise by measuring the plasma enrichment of infused isotopically labeled amino acids. Acute resistance exercise significantly altered whole-body amino acid kinetics (main effects of exercise *p* < 0.05) in both groups (n3-PUFA and placebo) on both inpatient study days (baseline, follow-up; Figure 4a–e). Because all measurements were performed during isotopic steady state, the flux of phenylalanine (Figure 4a) and tyrosine (Figure 4b) are equal to both the rates of appearance and disappearance of these amino acids [56]. Phenylalanine and tyrosine flux were significantly lower after acute resistance exercise. The conversion of phenylalanine to tyrosine represents the oxidation of phenylalanine and was also reduced following acute exercise (Figure 4c). The synthesis of protein from phenylalanine (Figure 4d) was determined by subtracting the rate of oxidation of phenylalanine from the phenylalanine rate of disappearance (phe flux). The balance between protein synthesis and protein breakdown (phenylalanine rate of appearance/phe flux) was also determined (Figure 4e). Because all measurements of amino acid kinetics were performed in the post-absorptive state, the rate of protein breakdown exceeded the rate of protein synthesis [56]. While whole-body protein synthesis was significantly lower post-exercise (Figure 4d), the overall protein balance significantly increased post-exercise (Figure 4e).

Six months of supplementation with n3-PUFA did not significantly alter the whole-body amino acid kinetic response to exercise (Figure 4a–e). In the conversion of phenylalanine to protein, we found significant main effects for both exercise (F = 95.055, *p* < 0.001) and time (F = 4.197, *p* = 0.046), as well as a significant time-by-exercise interaction (F = 4.781, *p* = 0.034; Figure 4d). This is also demonstrated in the difference in protein synthesis between pre- and post-exercise (Δ phe→protein; main effect of time F = 4.781, *p* = 0.034). The difference between pre and post exercise whole-body protein synthesis (Δ phe→protein) was smaller at follow-up, and post hoc pairwise comparisons indicated that it was only significantly different from baseline in the n3-PUFA group (*p* = 0.019), though the time-by-intervention interaction was not significant (F = 2.083, *p* = 0.156).

In addition to whole-body measurements of protein synthesis, muscle fractional synthesis rate (FSR) was also determined by measuring the incorporation of isotopically labelled amino acids into muscle protein. FSR was calculated from the change in isotopic enrichment in sequential muscle biopsies performed immediately prior to exercise and ~3 hrs after acute resistance exercise (Figure 1c). The isotopic enrichment in the plasma was used as the precursor pool to determine the lower limit of the true FSR, and the isotopic enrichment in muscle tissue fluid was used as to determine the upper limit. For both the lower and upper limits of FSR, main effects of time were observed (F = 20.638, *p* < 0.001; F = 19.084, *p* < 0.001), with higher FSRs at follow-up compared to baseline (Figure 4f) in both groups. However, a significant intervention-by-time interaction was observed for FSR calculated from tissue fluid enrichments (F = 4.522, *p* = 0.040), and a trend towards a significant interaction was observed for the FSR calculated from plasma enrichments (F = 3.352, *p* = 0.075). Post hoc pairwise comparisons revealed that both the lower and upper limits of FSR increased significantly only in the placebo group, and not in the n3-PUFA group (Figure 4f). Taken together, the amino acid kinetic and FSR data indicate a modest attenuation in the acute exercise response following the n3-PUFA intervention.

## 4. Discussion

The compromised skeletal muscle mass and function that accompanies aging has serious and significant metabolic and functional implications and is associated with increased risk of falls, frailty, and all-cause mortality. Lifestyle interventions such as exercise can improve and slow the decline in muscle dysfunction but are often less efficacious in older adults compared to young adults. At a molecular and cellular level, aging is associated with an attenuated anabolic response to exercise and nutritional stimuli. Efforts to understand the mechanisms contributing to age-related anabolic resistance point to chronic inflammation as a potential factor. Consistent with that notion, early evidence indicates that n3-PUFA supplementation may enhance anabolic response to acute exercise [5] and adaptations to exercise training [33]. Here, we employed a rigorous randomized placebo-controlled double-blind clinical trial to further evaluate the potential use of n3-PUFAs to enhance skeletal muscle health in healthy older adults. We evaluated the effects of 6 months of n3-PUFA supplementation on muscle performance, mitochondrial function, exercise-induced changes in whole-body amino acid kinetics, and skeletal muscle anabolic response to exercise. The main findings are that 6 months of n3-PUFA supplementation modestly increased skeletal muscle strength in the absence of any clear effects on whole-body or muscle-specific protein metabolism or mitochondrial function.

Muscle weakness is associated with higher mortality in older adults, and maintaining muscle strength is vital for maintaining physical function and mobility. Annual losses in muscle strength outpace losses in muscle mass. On average, older adults aged 70–79 lose ~1% leg lean muscle mass per year, while annual losses in muscle strength average between 2.65% and 4.12%, depending on sex and race [61]. Based on these data, we would expect approximately 0.5% loss in leg lean mass over the course of the 6-month intervention. A recent meta-analysis of the effects of n3-PUFA supplementation on muscle mass and strength in older adults reported consensus increases in muscle mass with a small to moderate effect size, but no consensus increases in muscle strength [62]. In the present study, the average changes in leg lean mass over 6 months were −0.01 kg (0.07%) for the placebo group and +0.07 kg (0.52%) for the n3-PUFA group. However, it should be noted that DEXA-based measurements of lean mass are not ideal for detecting small changes over a 6-month period in healthy older adults. Similarly, instead of the predicted 1.5–2.0% loss in muscle strength over 6 months, we observed an increase in single-leg leg extension 1-RM in both groups. Despite habituation and familiarization, this increase in strength may be attributable to a learning effect with the leg extension protocol, as individuals naïve to resistance exercise show greater variability and increases in 1-RM between initial testing sessions compared to experienced exercisers [63]. However, despite both groups showing increased strength at follow-up, this increase in strength was significant only in the n3-PUFA group (7.5 ± 14.1% increase), and not the placebo group (3.1 ± 14.7%). This increase appeared to be driven by improved muscle quality, as strength normalized to leg lean mass also increased in the n3-PUFA group. There is ambiguity regarding the effects of n3-PUFA supplementation on muscle performance. While some have reported increased strength with n3-PUFA supplementation [64,65], others have not [66,67]. Variable results could be due to study differences in testing protocols, n3-PUFA dosage and composition, and habitual activity levels. In a cohort of older adults with low muscle mass, n3-PUFA supplementation did not improve grip strength [66]. However, in line with our findings, healthy, independent-living older adults have shown improved grip strength and 1-RM composite strength scores following n3-PUFA supplementation [64]. The added benefit of combining n3-PUFA supplementation with exercise training interventions is also somewhat unclear. Some reports fail to see any additional benefits of n3-PUFA beyond those associated with resistance training alone [68], while others report greater improvements in strength and functional capacity when resistance training is combined with n3-PUFA supplementation [33,69]. Interestingly, in older adults, daily administration of the cyclooxygenase inhibitor acetaminophen enhanced resistance training adaptations, particularly in Type I fibers [70]. In the present study, muscle strength, but not power, was increased with n3-PUFA supplementation, suggesting that the beneficial effects of n3-PUFAs on muscle strength may be mediated by their COX-inhibiting properties [71]. Overall, the new data from the current study indicate that n3-PUFA supplementation results in modest, but meaningful gains in muscle strength in healthy older adults.

The potential for n3-PUFA supplementation to augment the strength gains associated with exercise training by enhancing the acute anabolic response has been supported by reports by our group [5] and others [26]. In this study, we examined the effects of n3-PUFA on the acute anabolic response to exercise in skeletal muscle and at the whole-body level. In our previous work, both basal and post-exercise mixed muscle protein FSR were elevated after the n3-PUFA intervention [5]. In the present study, exercise-associated FSR increased following 6 months of n3-PUFA supplementation, but this occurred to a similar, and potentially greater, extent in the placebo group. Our previous study did not include a placebo group, and the finding that FSR increased at follow-up in both groups in the present study highlights the importance of the placebo control in the interpretation of time-dependent changes. It is possible that the increased FSR in both groups reflects familiarization with the exercise and the recruitment of a greater number of muscle fibers [72], though the absolute workload (repetitions × weight) during the inpatient study visit did not differ significantly between baseline and follow-up. Interestingly, despite an overall effect of time on FSR in both groups, the post-intervention increase in FSR was attenuated in the n3-PUFA group. In our previous work, basal postabsorptive FSR was measured prior to exercise, and post-exercise FSR was evaluated 15–18 h after acute exercise, at which point FSR is predicted to be elevated [73]. An important distinction is that the current study measured muscle protein synthesis over a single ~4-h time period that included the exercise bout and an early (~3 h) recovery period. Inasmuch, we cannot ignore the possibility that the attenuation of muscle FSR response to acute exercise with n3-PUFA supplementation could reflect reduced physiological stress in the muscle.

At the whole-body level, n3-PUFA supplementation also exhibited a modest attenuating effect on post-exercise whole-body protein synthesis. In both groups and at both time points, acute exercise reduced the rate of protein synthesis (Phe→Protein). In the post-absorptive state, amino acids may be used as a fuel source rather than for protein synthesis during exercise and in the recovery period [74], which may account for the reduced protein synthesis following exercise. As with muscle-specific FSR, exercise-induced alterations in the whole-body conversion of phenylalanine to protein were altered after the intervention in both groups. While whole-body protein synthesis was lower after acute exercise, at follow-up the change in whole-body protein synthesis after exercise was significantly smaller (i.e., the Δ Phe→Protein from pre- to post-exercise was closer to 0) only in the n3-PUFA group. As may be hypothesized with the FSR, the blunted exercise response in protein synthesis in the n3-PUFA group may also indicate a decrease in the physiological stress associated with acute exercise.

It is also worth considering the possibility that the anti-inflammatory effects of n3-PUFA may blunt the acute anabolic responses to exercise in individuals without evidence of chronic inflammation. High-dose over-the-counter non-steroidal anti-inflammatory drugs have been shown to attenuate strength and volume gains in muscle mass in healthy young adults [75]. However, in older adults, these same drugs have been shown to enhance adaptations to resistance exercise training [25]. Although exercise training leads to reduced systemic inflammation [76], acute exercise leads to short-term inflammatory responses that include cytokine signaling and immune cell activity that are important for repair processes [77,78]. It is conceivable that attenuating pro-inflammatory pathways in healthy individuals in the absence of chronic inflammation could interfere with appropriate post-exercise signaling pathways, while having the opposite effect in individuals with heightened inflammatory pathways at rest. In individuals with heightened levels of inflammation, it is also possible that anti-inflammatory agents attenuate the acute response to exercise, while enhancing long-term adaptations.

Mitochondria are critical determinants of skeletal muscle health because of their role in supporting the energetic demands of muscle contraction and cellular processes, signaling through reactive oxygen species, and regulating autophagy. In preclinical studies, we found that dietary fish oil restored mitochondrial oxidative capacity in aged mice [34]. In an early human trial, we reported lower mitochondrial ROS emissions in older adults following n3-PUFA supplementation [5]. In contrast with these early findings, in the current study mitochondrial function was unchanged following the intervention. Aging is commonly associated with decreased mitochondrial protein content as well as dysfunction at the level of the organelle [79], so it is worth noting that this cohort of older adults did not demonstrate the typical age-related deficits in mitochondrial function [39]. Therefore, it is important to not generalize these results beyond the situation of healthy aging since we cannot rule out the possibility that n3-PUFA may enhance mitochondrial energy metabolism in other aging populations that exhibit more profound sarcopenia, frailty, and dysfunctional mitochondria. In addition, while assessing mitochondrial function in permeabilized fibers provides physiologically relevant data at the tissue-level, it does not provide information regarding mitochondrial content or function at the level of the organelle.

In summary, we found that 6 months of n3-PUFA supplementation modestly increased skeletal muscle strength in the absence of any measurable change in mitochondrial physiology. Notably, this cohort of older adults was healthy, independent and capable of performing activities of daily living, and did not exhibit the typical features of frailty or chronic inflammation. Future research may elucidate whether n3-PUFA treatment may provide greater therapeutic benefits to skeletal muscle in older adults with skeletal muscle functional impairments, chronic inflammation, sarcopenia or metabolic disease.

## Figures and Tables

**Table 1:** Participant characteristics and body composition before and after 6 months of placebo or n3-PUFA supplementation.

	Placebo (*n* = 33) Mean (SD)		n3-PUFA (*n* = 30) Mean (SD)		Group Effect Statistic (*p*-Value)	Time Effect Statistic (*p*-Value)	Interaction Statistic (*p*-Value)
	Baseline	Follow-Up	Baseline	Follow-Up			
Sex	20F/13M		14F/16M				
Age (years)	71.5 (4.8)		71.4 (4.5)		0.100 (0.920)		
Height (cm)	168.1 ± 10.1		168.6 ± 8.9		−0.208 (0.836)		
Weight (kg)	74.5 (14.5)	74.5 (14.5)	77.3 (13.1)	77.1 (13.6)	0.592 (0.445)	0.148 (0.702)	0.145 (0.705)
BMI (kg/m^2^)	26.3 (3.9)	26.3 (3.8)	27.1 (4.0)	27.2 (4.3)	0.718 (0.400)	0.178 (0.675)	0.040 (0.841)
SMI (kg/m^2^)	7.0 (1.3)	7.0 (1.3)	7.2 (1.0)	7.2 (1.1)	0.241 (0.625)	0.747 (0.391)	0.020 (0.889)
Body Fat (%)	35.8 (8.9)	35.8 (9.1)	36.9 (7.3)	36.7 (7.2)	0.228 (0.635)	0.075 (0.785)	0.176 (0.677)
Lean Mass (kg)	45.5 (10.6)	45.5 (10.5)	46.0 (8.0)	46.1 (8.1)	0.058 (0.811)	0.008 (0.929)	0.002 (0.957)
Leg Lean Mass (kg)	15.2 (3.9)	15.2 (3.8)	15.3 (3.0)	15.4 (3.1)	0.057 (0.813)	0.225 (0.637)	0.302 (0.585)
RBC EPA (µM)	7.8 (4.2)	5.1 (1.5) *	6.2 (4.9) †	37.1 (12.4) †*	**94.845 (<0.001)**	**145.908 (<0.001)**	**302.957 (<0.001)**
RBC DHA (µM)	69.4 (23.3)	57.0 (13.0) *	58.0 (25.5) †	88.7 (17.4) †*	3.958 (0.051)	**15.171 (<0.001)**	**69.690 (<0.001)**
ESR (mm/h)	8.8 (6.5)	10.1 (9.3)	7.8 (6.2)	7.5 (6.2)	0.753 (0.389)	0.053 (0.818)	0.504 (0.480)
CRP (mg/L)	1.6 (1.3)	2.2 (2.5)	1.7 (1.8)	1.5 (1.9)	0.373 (0.543)	0.028 (0.867)	0.940 (0.336)

BMI: body mass index; SMI: skeletal muscle index; RBC: red blood cell; EPA: eicosapentaenoic acid; DHA: docosahexanoic acid; ESR: erythrocyte sedimentation rate; CRP: *C*-reactive protein. Unpaired *t*-tests were performed to assess differences between groups in age and height. Mixed repeated measures ANOVAs were performed to determine the within-subjects effects of time (baseline, follow-up), the between-subjects effects of the intervention (n3-PUFA, placebo) and the interaction between time and intervention. Post hoc pairwise comparisons were performed when significant main or interaction effects were identified. Significance was set a priori at *p* < 0.05. * represents a significant (*p* < 0.05) within-group difference from baseline; † represents a significant (*p* < 0.05) difference from placebo at the corresponding time point; bold values represent significant main or interaction effects.

## Data Availability

The data presented in this study are available on request from the corresponding author.
